# Mechanical thrombectomy for massive PE with incidental discovery of a patent foramen ovale: a case report

**DOI:** 10.1186/s42155-026-00659-x

**Published:** 2026-02-03

**Authors:** Alameen Damer, Alfredo Páez-Carpio, Christian Houbois, Gilbert Maroun

**Affiliations:** 1 University of Toronto Department of Medical Imaging, Toronto, Canada; 2 Sunnybrook Health Sciences Centre, Department of Medical Imaging, Toronto, Canada

To the editor,

Pulmonary embolism (PE) affects 60–120 individuals per 100,000, with significant morbidity/mortality [1–3]. The European Society of Cardiology (ESC) guidelines stratify risk by hemodynamics, RV dysfunction, and troponin, guiding therapy from medical to surgical/endovascular embolectomy [4]. Percutaneous catheter-directed treatments such as aspiration and mechanical thrombectomy improve thrombus burden, right ventricle (RV) function, hemodynamics, and outcomes in intermediate and high-risk PE [4–7]. Unrecognized patent foramen ovale (PFO), found in up to 25% of adults, poses unique risks during thrombectomy [8, 9]. Prior reports have described adverse events in the context of PFOs, mainly paradoxical embolism. This report will expand on the unique risks associated with thrombectomy in the context of a PFO, namely the risk of undetected left-sided entry [10, 11].

We present an 85-year-old woman with tachycardia (109 beats per minute), tachypnea (30 breaths per minute), reduced consciousness, elevated troponin (147 ng/mL), and lactate (7.0 mmol/L). She deteriorated to refractory hypoxemia requiring intubation and vasopressors (norepinephrine 0.02 μg/kg/min; vasopressin 0.03 units/min) to maintain her systolic pressures in the 90 s. CT pulmonary angiography showed extensive bilateral pulmonary emboli and severe right heart strain (RV/left ventricle (LV) ratio 1.53) (Fig. 1). The main, right, and left pulmonary arteries were normal. The lungs and pleura were normal, and no intracardiac shunt or PFO were identified on this pre-procedural CT scan. Classified as high-risk PE, she underwent urgent catheter-directed thrombectomy without pre-procedural echocardiography due to hemodynamic instability.

**Fig. 1:**
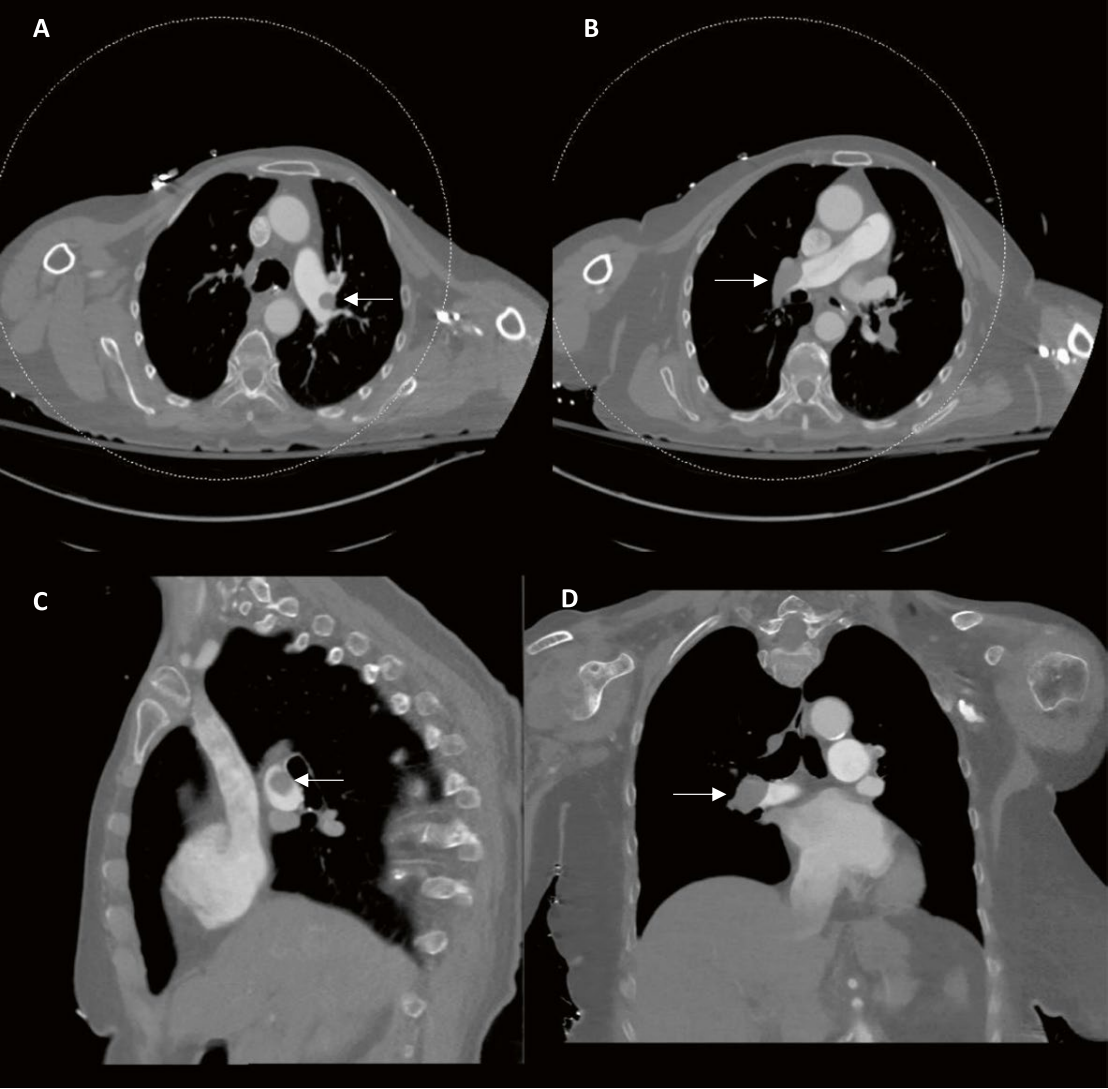
Multiplanar CT images demonstrating bilateral pulmonary arterial filling defects (white arrows)

The patient was already intubated and sedated with 75-μg fentanyl and 25-mg intravenous ketamine prior to and during the procedure. Right femoral venous access was obtained with a 0.018″ micropuncture system, and a 6 French vascular sheath (Terumo®, Tokyo, Japan) was placed. A 0.035″ Glidewire and 5 French angled pigtail catheter (Merit Medical, South Jordan, UT, USA) were advanced past the cavoatrial junction under the assumption of normal right-sided anatomy. While attempting to cannulate the pulmonary artery, the catheter entered a vessel inferior to the carina, an unexpected course. Contrast injection opacified a pulmonary vein. The catheter was withdrawn to the atrial level, and contrast opacified the aorta (Fig. 2). Further withdrawal into the inferior vena cava (IVC) showed early left-sided opacification, again consistent with shunting (Fig. 3). Re-advancement using a 5 French Glidecath C2 catheter (Terumo) allowed for maneuvering into the true right atrium at an acute angle of entry and subsequently the right ventricle, which was confirmed with contrast injections. The PFO caused preferential left atrial passage, requiring deliberate manipulation and an acute angle to access the right heart (Fig. 4).

**Fig. 2:**
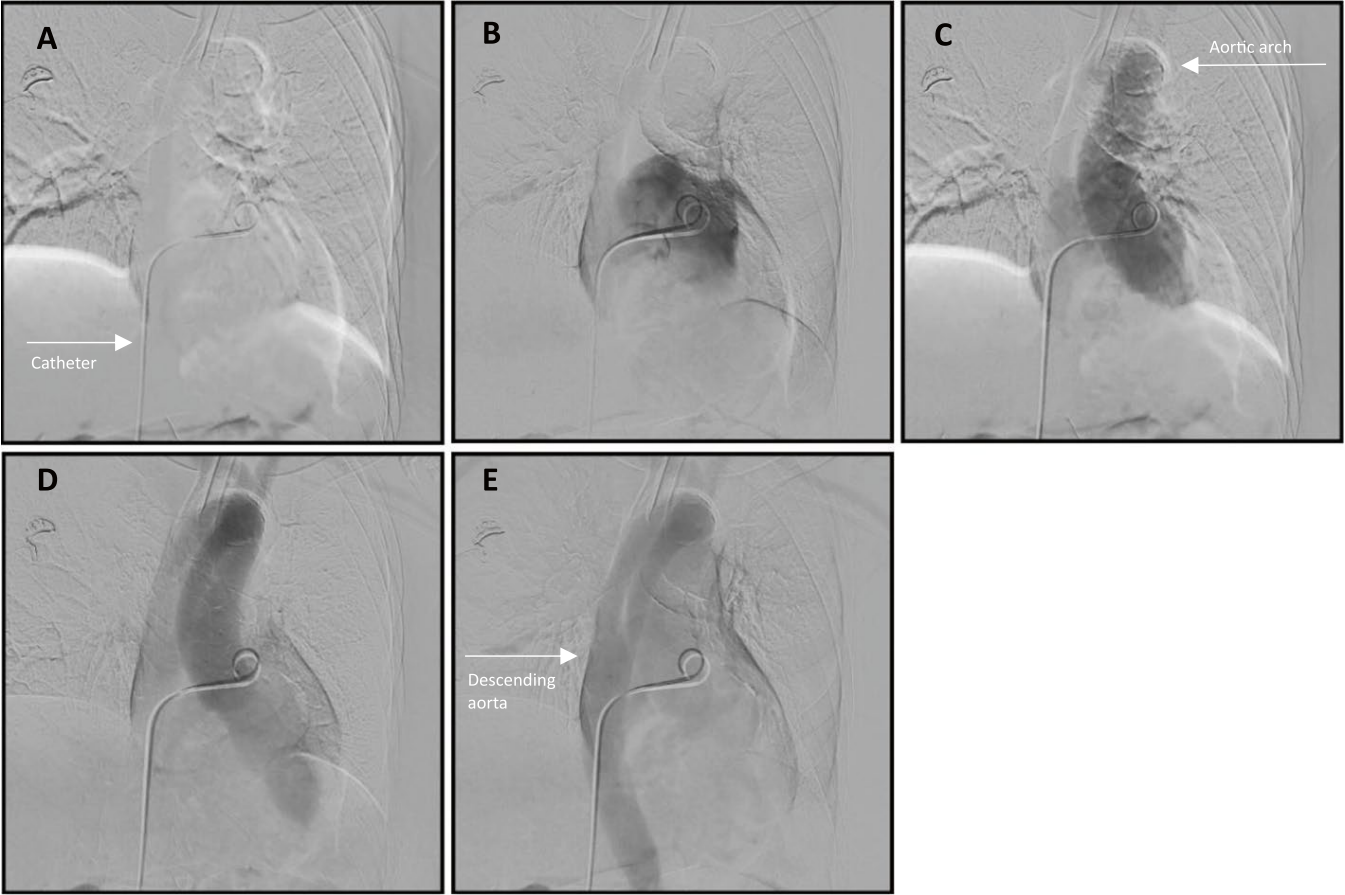
**A** The catheter is advanced through the IVC and past the inferior cavoatrial junction. **B** Contrast is administered. **C**–**E** Progressive opacification of the aorta is noted

**Fig. 3:**
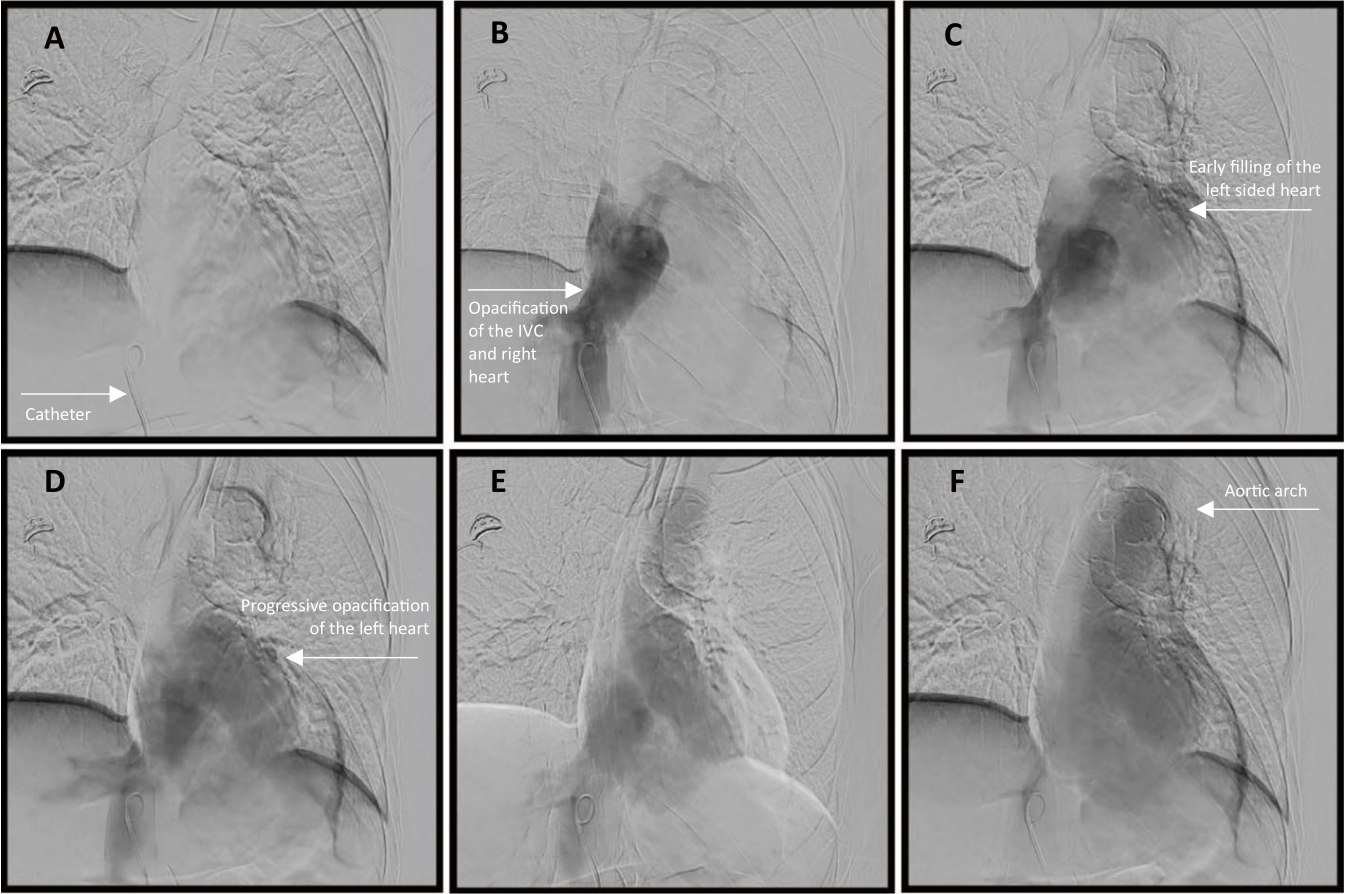
**A** The catheter is retracted back to the IVC. **B** Contrast is administered. **C**–**F** There is early opacification of the left of the heart and aortic arch, further raising suspicion for a PFO

**Fig. 4:**
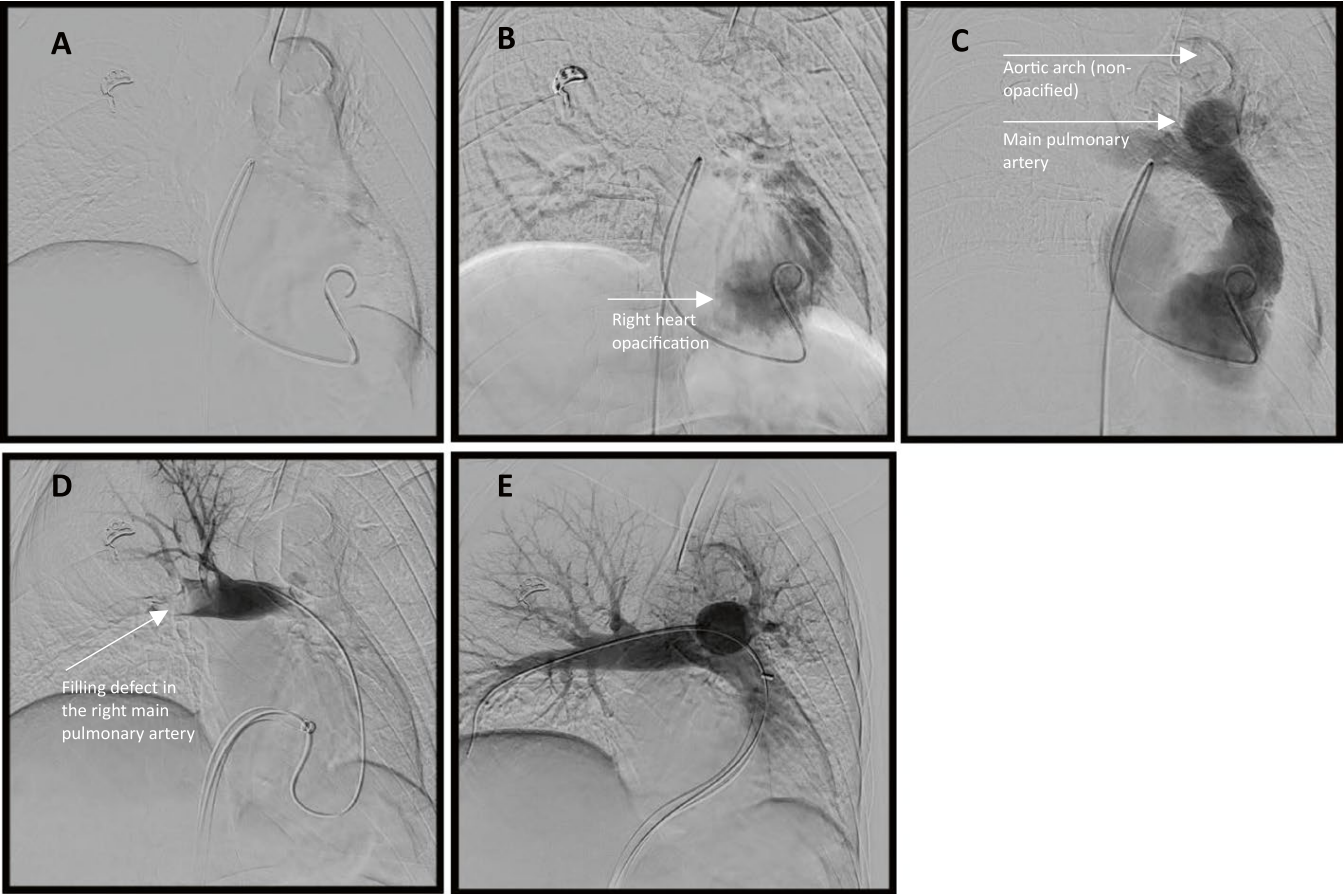
**A**–**C** The catheter is maneuvered into the right atrium, which requires an acute angle of entry. **D** Representative image after contrast administration demonstrated filling defects in the right main pulmonary artery (bottom left). **E** Post-thrombectomy images show no residual filling defects (bottom center)

Selective catheterization of the pulmonary trunk and branches was achieved using a combination of the previously used 5 French angled pigtail, Glidecath C2, and DAV (Cook Medical®, Bloomington, IN, USA) catheters. Ten milligram of tissue plasminogen activator (tPA) was administered bilaterally. A 24 French FlowTriever sheath was advanced into the IVC and then a 24 French FlowTriever aspiration catheter (Inari Medical®, Irvine CA, USA) was advanced into the pulmonary arteries over an extra-stiff Amplatz wire (Boston Scientific®, Marlborough, MA, USA), and aspiration retrieved significant clot burden. Post-procedure angiography demonstrated satisfactory clearance (Fig. 4). An Inari FlowStasis device was deployed for hemostasis, with minimal estimated blood loss, and the procedure concluded without acute complication.

The patient was extubated the next morning, vasopressors discontinued, and hemodynamics stabilized over 2 weeks (blood pressure of 108/79, heart rate of 87, oxygen saturation of 98% room air). An echocardiogram showed normal RV, hyperdynamic LV, and small PFO. Follow-up CT confirmed PE resolution and no lung disease. Of note, approximately 1 week post procedure, she developed vision and cognitive deficits that prompted a CT scan which revealed a large left posterior cerebral artery (PCA) infarct, likely paradoxical embolism.

She remains stable from a cardiorespiratory perspective, with ongoing functional improvement but persistent cognitive and visual deficits.

Prior reports primarily describe embolic sequelae associated with PFOs after thrombectomy. Uecker et al. described paradoxical embolization causing right M2 occlusion and left-sided neglect [10]. Nezami et al. reviewed 9 cases, noting one left middle cerebral artery (MCA) infarct post-intervention [11]. Although paradoxical embolism occurred, our case highlights the procedural risk of left-sided entry and inadvertent advancement of large-bore devices into the left heart.

Pulmonary veins are smaller in caliber than pulmonary arteries, measuring approximately 9–18 mm compared with pulmonary artery diameters of up to 22 mm [12–14]. Advancement of large-bore catheters into these smaller vessels risks injury or rupture, which may be fatal.

This case highlights several preventive strategies for operators. Routine intraprocedural confirmation of chamber and vessel position using contrast or limited angiography should be performed at each major advancement. Advancing guidewires and smaller catheters before large-bore devices provides an added safeguard, particularly when a PFO has not been excluded. Operators should also maintain a high index of suspicion for atypical catheter trajectories, such as courses inferior to the carina or unexpected vessel caliber, prompting immediate contrast confirmation. Waveform analysis of the cannulated vessel may theoretically aid localization, as pulmonary artery waveforms differ from pulmonary veins, but this approach has not been validated in PE thrombectomy and warrants further study [15, 16].

When clinically feasible (e.g., hemodynamic stability and timely access), transthoracic or transesophageal echocardiography may be considered prior to intervention. However, patients requiring percutaneous thrombectomy are often intermediate–high or high risk with elevated troponin and RV dysfunction, and thrombectomy should not be delayed for echocardiography [4].

In conclusion, operators must remain vigilant for unrecognized PFOs, which may hinder procedures and cause significant harm if not considered.

## Data Availability

No applicable.
